# Viral Population Estimation Using Pyrosequencing

**DOI:** 10.1371/journal.pcbi.1000074

**Published:** 2008-05-09

**Authors:** Nicholas Eriksson, Lior Pachter, Yumi Mitsuya, Soo-Yon Rhee, Chunlin Wang, Baback Gharizadeh, Mostafa Ronaghi, Robert W. Shafer, Niko Beerenwinkel

**Affiliations:** 1 Department of Statistics, University of Chicago, Chicago, Illinois, United States of America; 2 Department of Mathematics, University of California, Berkeley, California, United States of America; 3 Division of Infectious Diseases, Stanford University Medical Center, Stanford, California, United States of America; 4 Genome Technology Center, Stanford University, Palo Alto, California, United States of America; 5 Department of Biosystems Science and Engineering, ETH Zurich, Basel, Switzerland; University of California San Diego, United States of America

## Abstract

The diversity of virus populations within single infected hosts presents a major difficulty for the natural immune response as well as for vaccine design and antiviral drug therapy. Recently developed pyrophosphate-based sequencing technologies (pyrosequencing) can be used for quantifying this diversity by ultra-deep sequencing of virus samples. We present computational methods for the analysis of such sequence data and apply these techniques to pyrosequencing data obtained from HIV populations within patients harboring drug-resistant virus strains. Our main result is the estimation of the population structure of the sample from the pyrosequencing reads. This inference is based on a statistical approach to error correction, followed by a combinatorial algorithm for constructing a minimal set of haplotypes that explain the data. Using this set of explaining haplotypes, we apply a statistical model to infer the frequencies of the haplotypes in the population via an expectation–maximization (EM) algorithm. We demonstrate that pyrosequencing reads allow for effective population reconstruction by extensive simulations and by comparison to 165 sequences obtained directly from clonal sequencing of four independent, diverse HIV populations. Thus, pyrosequencing can be used for cost-effective estimation of the structure of virus populations, promising new insights into viral evolutionary dynamics and disease control strategies.

## Author Summary

The genetic diversity of viral populations is important for biomedical problems such as disease progression, vaccine design, and drug resistance, yet it is not generally well understood. In this paper, we use pyrosequencing, a novel DNA sequencing technique, to reconstruct viral populations. Pyrosequencing produces DNA sequences, called reads, in numbers much greater than standard DNA sequencing techniques. However, these reads are substantially shorter and more error-prone than those obtained from standard sequencing techniques. Therefore, pyrosequencing data requires new methods of analysis. Here, we develop mathematical and statistical tools for reconstructing viral populations using pyrosequencing. To this end, we show how to correct errors in the reads and assemble them into the different viral strains present in the population. We apply these methods to HIV-1 populations from drug-resistant patients and show that our techniques produce results quite close to accepted techniques at a lower cost and potentially higher resolution.

## Introduction

Pyrosequencing is a novel experimental technique for determining the sequence of DNA bases in a genome [1],[2]. The method is faster, less laborious, and cheaper than existing technologies, but pyrosequencing reads are also significantly shorter and more error-prone (about 100–250 base pairs and 5–10 errors/kb) than those obtained from Sanger sequencing (about 1000 base pairs and 0.01 errors/kb) [3]–[5].

In this paper we address computational issues that arise in applying this technology to the sequencing of an RNA virus sample. Within-host RNA virus populations consist of different haplotypes (or strains) that are evolutionarily related. The population can exhibit a high degree of genetic diversity and is often referred to as a quasispecies, a concept that originally described a mutation-selection balance [6],[7]. Viral genetic diversity is a key factor in disease progression [8],[9], vaccine design [10],[11], and antiretroviral drug therapy [12],[13]. Ultra-deep sequencing of mixed virus samples is a promising approach to quantifying this diversity and to resolving the viral population structure [14]–[16].

Pyrosequencing of a virus population produces many reads, each of which originates from exactly one—but unknown—haplotype in the population. Thus, the central problem is to reconstruct from the read data the set of possible haplotypes that is consistent with the observed reads and to infer the structure of the population, i.e., the relative frequency of each haplotype.

**Figure 1:**
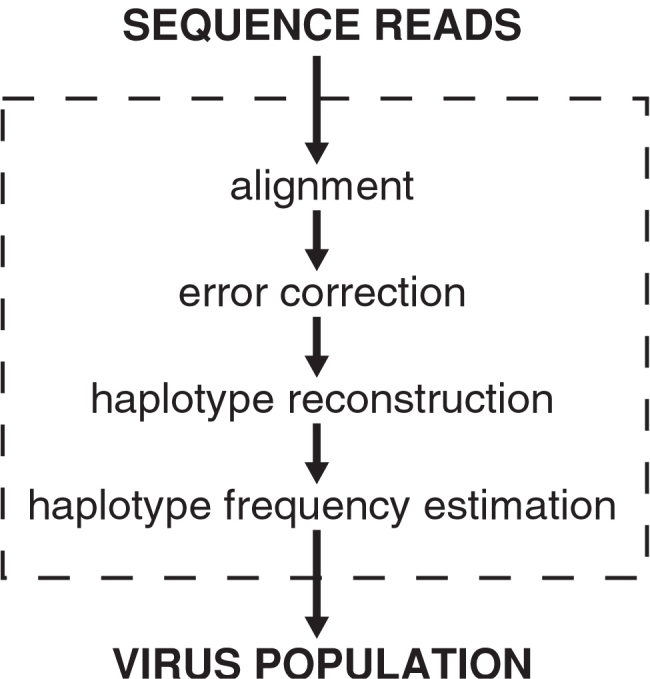
Overview of viral population estimation using pyrosequencing. Sequence reads are first aligned to a reference strain, then corrected for errors, and assembled into haplotype candidates. Finally, the relative frequencies of the reconstructed haplotypes are estimated in a ML fashion. These estimates constitute the inferred virus population.

Here we present a computational four-step procedure for making inference about the virus population based on a set of pyrosequencing reads (Figure 1). First, the reads are aligned to a reference genome. Second, sequencing errors are corrected locally in windows along the multiple alignment using clustering techniques. Next, we assemble haplotypes that are consistent with the observed reads. We formulate this problem as a search for a set of covering paths in a directed acyclic graph and show how the search problem can be solved very efficiently. Finally, we introduce a statistical model that mimics the sequencing process and we employ the maximum likelihood (ML) principle for estimating the frequency of each haplotype in the population.

The alignment step of the proposed procedure is straightforward for the data analyzed here and has been discussed elsewhere [5]. Due to the presence of a reference genome, only pair-wise alignment is necessary between each read and the reference genome. We will therefore focus on the core methods of error correction, haplotype reconstruction, and haplotype frequency estimation. Two independent approaches are pursued for validating the proposed method. First, we present extensive simulation results of all the steps in the method. Second, we validate the procedure by reconstructing four independent HIV populations from pyrosequencing reads and comparing these populations to the results of clonal Sanger sequencing from the same samples.

These datasets consist of approximately 5000 to 8000 reads of average length 105 bp sequenced from a 1 kb region of the *pol* gene from clinical samples of HIV-1 populations. Pyrosequencing (with the Roche GS20 platform [17]) can produce up to 200,000 usable reads in a single run. Part of our contribution is an analysis of the interaction between the number of reads, the sequencing error rate and the theoretical resolution of haplotype reconstruction. The methods developed in this paper scale to these huge datasets under reasonable assumptions. However, we concentrate mainly on a sample size (about 10,000 reads) that produces finer resolution than what is typically obtained using limiting dilution clonal sequencing. Since many samples can be run simultaneously and independently, this raises the possibility of obtaining data from about 20 populations with one pyrosequencing run.

Estimating the viral population structure from a set of reads is, in general, an extremely hard computational problem because of the huge number of possible haplotypes. The decoupling of error correction, haplotype reconstruction, and haplotype frequency estimation breaks this problem into three smaller and more manageable tasks, each of which is also of interest in its own right. The presented methods are not restricted to RNA virus populations, but apply whenever a reference genome is available for aligning the reads, the read coverage is sufficient, and the genetic distance between haplotypes is large enough. Clonal data indicates that the typical variation in the HIV *pol* gene is about 3 to 5% in a single patient [18]. We find that as populations grow more diverse, they become easier to reconstruct. Even at 3% diversity, we find that much of the population can be reconstructed using our methods.

The *pol* gene has been sequenced extensively and (essentially) only one specific insertion seems to occur, namely the 69 insertion complex, which occurs under NRTI pressure [19]. None of our samples were treated with NRTIs, and the Sanger clones did not display this (nor any other) indel. Therefore we assume throughout that there are no true indels in the population. However, the algorithms developed in this paper generalize in a straightforward manner for the case of true indels.

The problem of estimating the population structure from sequence reads is similar to assembly of a highly repetitive genome [20]. However, rather than reconstructing one genome, we seek to reconstruct a population of very similar genomes. As such, the problem is also related to environmental sequencing projects, which try to assess the genomes of all species in a community [21]. While the associated computational biology problems are related to those that appear in other metagenomics projects [22], novel approaches are required to deal with the short and error-prone pyrosequencing reads and the complex structure of viral populations. The problem is also similar to the haplotype reconstruction problem [23], with the main difference being that the number of haplotypes is unknown in advance, and to estimating the diversity of alternative splicing [24].

More generally, the problem of estimating diversity in a population from genome sequence samples has been studied extensively for microbial populations. For example, the spectrum of contig lengths has been used to estimate diversity from shotgun sequencing data [25]. Using pyrosequencing reads, microbial diversity has been assessed by counting BLAST hits in sequence databases [26]. Our methods differ from previous work in that we show how to analyze highly directed, ultra-deep sequencing data using a rigorous mathematical and statistical framework.

## Results

We have developed a computational and statistical procedure for inferring the structure of a diverse virus population from pyrosequencing reads. Our approach comprises four consecutive steps (Figure 1), starting with the alignment of reads to a reference sequence and followed by error correction, haplotype reconstruction, and haplotype frequency estimation.

### Error correction

Given the high error rate of pyrosequencing, error correction is a necessary starting point for inferring the virus population. The errors in pyrosequencing reads typically take the form of one-base indels along with substitutions and ambiguous bases and occur most often in homopolymeric regions. The reads come with quality scores for each base quantifying the probability that the base is correct.

Error rates with the Roche GS20 system have been estimated as approximately 5 to 10 errors per kb [4],[5]. However, a small number of reads accounts for most of the errors. Thus after discarding approximately 10% of the reads (those with ambiguous bases or atypical length), the error can be reduced to 1 to 3 errors per kb [4]. These remaining errors are about half insertions and a quarter each deletions and substitutions.

Due to our assumption that there are no haplotypes with insertions in the population, the insertions in the reads can all be simply corrected through alignment with the reference genome. We do not deal with the problem of alignment here; it is straightforward because our assumption of the existence of a reference genome implies that only pair-wise alignment is necessary. In the remainder of the paper, we assume that a correct alignment is given, leaving about 1 error per kb to correct (or 3 errors per kb if the low-quality reads are not aggressively pruned).

**Figure 2:**
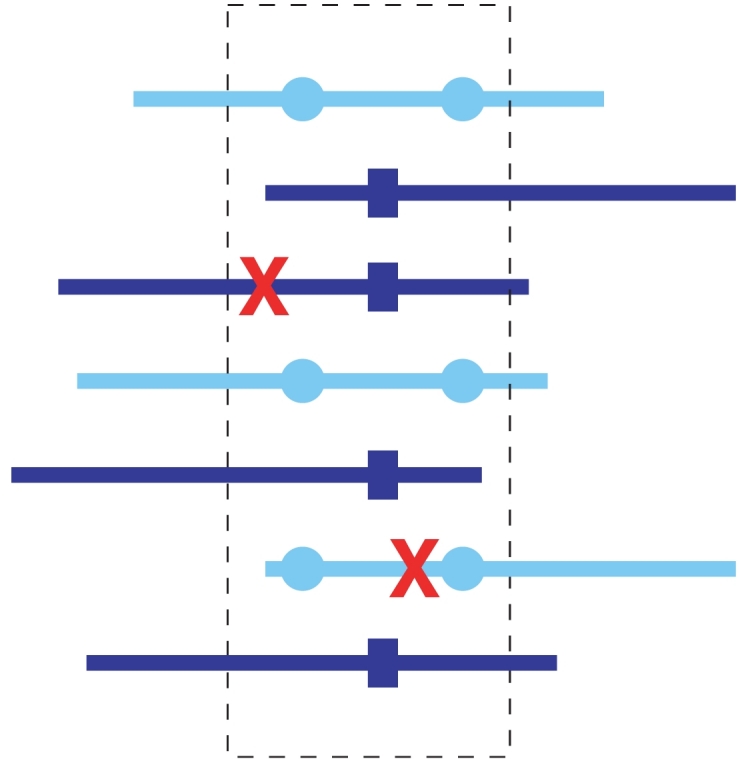
Error correction. Fixed-width windows (shown as the dashed box) over the aligned reads are considered. Two different types of reads are depicted (light versus dark lines), indicating their origin from two different haplotypes. Genetic differences (indicated by circles and squares) provide the basis for clustering reads into groups representing the respective haplotypes. After clustering, errors (marked as crosses) can be corrected.

Our approach for error correction resembles the method of [27] for distinguishing repeats in whole genome shotgun assemblies combined with [28]. We consider all the reads in a window over the multiple alignment and cluster these reads using a statistical testing procedure to detect if a group of reads should be further split (Figure 2). The reads in each cluster are then corrected to the consensus sequence of the cluster.

The statistical testing procedure consists of two steps. First, every column in the window is tested for over-representation of a mutation using a binomial test. Second, every pair of columns is tested to see if a pair of mutations happens together more often than would be expected by chance. See Methods for details on the tests.

Any significant over-representation of a mutation or a pair in a window is regarded as evidence for the reads originating from more than one haplotype. The testing procedure produces an estimate for the number of haplotypes in the window as follows. First, all single mutations are tested for significance; each significant mutation gives evidence for another haplotype in the window. Next, all pairs of mutations are tested; any significant pairs is evidence for another haplotype. However, this process can over-count the number of haplotypes in the window in certain cases if two mutations are significant both by themselves and as a pair. In this case, we correct for the over-count (see Methods).

We then separate the reads into *k* groups using *k*-means clustering. The algorithm is initialized with both random clusters and clusters found by a divisive clustering method based on the statistical tests. We use the Hamming distance between sequences to calculate cluster membership; the consensus sequences define the cluster centers. The consensus sequence is computed from weighted counts based on the quality scores. Thus, the inferred cluster centers are the reconstructed haplotypes. Combining testing and clustering we proceed as follows:

### Algorithm 1. (Local error correction)

Input: A window of aligned reads

Output: The *k* haplotypes in the window and the error corrected reads

Procedure:

1. Find all candidate mutations and pairs of mutations and test for overrepresentation.
2. Count the number of non-redundant mutations and pairs that are significant. This is the number *k* of haplotypes in the window.
3. Cluster the reads in the window into *k* clusters and correct each read to its cluster center.
4. Output corrected reads.

Applying a parsimony principle the algorithm finds the smallest number *k* of haplotypes that explain the observed reads in each window. The genomic region to be analyzed is divided into consecutive windows and Algorithm 1 is run in each of them. We use three collections of successive windows that are shifted relative to each other such that each base in the region is covered exactly three times. The final correction of each base is the consensus of the three runs.

The error correction procedure can lead to uncorrected errors or miscorrections via false positives and negatives (leading to over/underestimation of the number of haplotypes in a window) or misclustering. See Methods for implementation details and a discussion of setting the parameters so as to minimize these mistakes.

False positives arise when an error is seen as a significant variant; they will be consequences of setting the error rate too low or the significance level too high, or if errors are highly correlated. Misclustering can happen if errors occur frequently enough on a single read to make that read appear closer to an incorrect haplotype. This likelihood is increased as the window size grows and more reads overlap the window only partially.

An analysis of the false negative rate gives an idea of the theoretical resolution of pyrosequencing. False negatives arise when a true variant tests as non-significant and thus is erased. If the input data were error-free, this would be the only source of mistaken corrections and would happen by eliminating rare variants. Given an error rate of 2.5 errors per kb, the calculation in the Methods Section shows that variants present in under 1% of the population would be erased on a dataset of 10,000 reads. Below we will show that this number of reads is about enough to expect to resolve haplotypes present in 1% of the population.

### Haplotype reconstruction

Our approach to haplotype reconstruction rests on two basic beliefs. First, the haplotypes in the populations should not exhibit characteristics that are not present in the set of reads. This means that every haplotype in the population should be realizable as an overlapping series of reads. Second, the population should explain as many reads as possible with as few haplotypes as possible.

We assume a set **R** of aligned and error-corrected reads obtained from sequencing a population. If all haplotypes have the same length *n*, then each aligned read consists of a start position in the genome and a string representing the genomic sequence. We say that two reads overlap if there are positions in the genome to which they are both aligned. They agree on their overlap if they agree at all of these positions. We call a haplotype *completely consistent* with the set of reads **R** if the haplotype can be constructed from a subset of overlapping reads of **R** that agree on their overlaps. Let **C_R_** be the set of all haplotypes that are completely consistent with **R**. In the following, we provide methods for constructing and sampling from **C_R_** and we present an efficient algorithm for computing a lower bound on the number of haplotypes necessary to explain the reads. Both techniques rely on the concept of a read graph.

### Definition (Read graph)

The *read graph* **G_R_** associated with a set of reads **R** is the acyclic directed graph with vertices {**R**_irred_, *s*, *t*} consisting of a source *s*, a sink *t*, and one vertex for every irredundant read *r* ∈ **R**. Here, a read is *redundant* if there is another read that overlaps it completely such that the two reads agree on their overlap. The edge set of **G_R_** is defined by including an edge from an irredundant read *r*_1_ to an irredundant read *r*_2_, if

1. *r*_1_ starts before *r*_2_ in the genome,
2. *r*_1_ and *r*_2_ agree on their (non-empty) overlap, and
3. there would not be a path in **G_R_** from *r*_1_ to *r*_1_ without this edge.

Finally, edges are added from the source *s* to all reads beginning at position 1, and from all reads ending at position *n* to the sink *t*.

A path in the read graph from the source to the sink corresponds to a haplotype that is completely consistent with **R**. Thus, finding **C_R_**, the set of completely consistent haplotypes, amounts to efficiently enumerating paths in the read graph.

**Figure 3:**
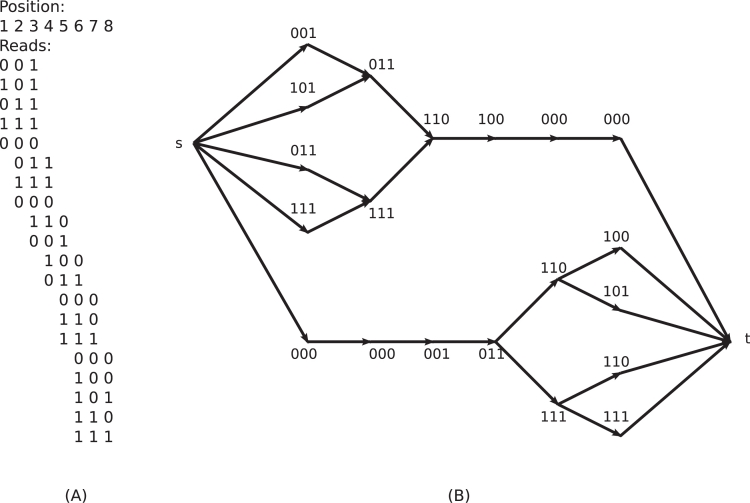
Read graph. A simplified genome of length *n* = 8 over the binary alphabet {0,1} is considered. Twenty reads of length 3 each are aligned to an assumed reference sequence (A). The induced read graph has 20+2 vertices and 28 edges (B).

**Figure 4:**
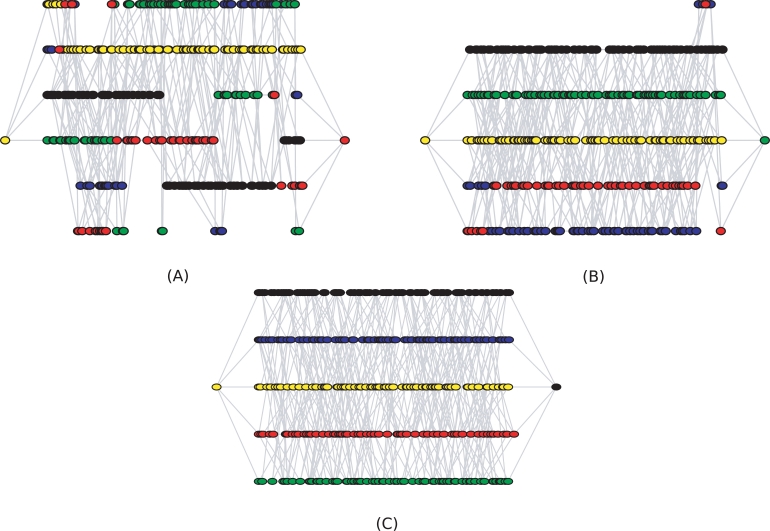
Read graphs for increasing diversity levels. Displayed are three read graphs of 1000 reads each derived from populations of 5 haplotypes at 3% (A), 5% (B), and 7% diversity (C). The bottom five lines in the graph correspond to reads which match the five haplotypes uniquely; the top line in subfigures (A) and (B) contains reads which match several haplotypes. In each subfigure, the reads are colored according to a single chain decomposition.

For example, in Figure 3, a simplified genome of length *n* = 8 over the binary alphabet {0,1} is considered, and an alignment of 20 reads, each of length 3, is shown in Figure 3A. These data give rise to the read graph depicted in Figure 3B. For instance, the haplotype 00110000 is completely consistent with the reads and corresponds to the top path in the graph. For more complex read graphs, see Figure 4.

We say that a set of haplotypes **H** is an *explaining* set for **R** if every read *r* ∈ **R** can be obtained as a substring of some haplotype in **H**. We seek a small set of explaining haplotypes and focus on the set **C_R_**, which consists exactly of those haplotypes that emerge from the data. The following proposition provides a criterion for **C_R_** to be an explaining set in terms of the read graph.

### Proposition

The set of haplotypes completely consistent with a set of reads is an explaining set for these reads if, and only if, every vertex of the read graph lies on a directed path from the source to the sink.

The Lander–Waterman model of sequencing is based on the assumptions that reads are random (uniformly distributed on a genome) and independent [29]. In this model, the probability that all bases of a genome of length *n* are sequenced follows the Poisson distribution *p* = (1−*e* ^−*c*^)*^n^*, where *c* is the coverage (the total number of bases sequenced per position). For a sequencing experiment from a mixed population with different abundances of haplotypes (or subspecies), a similar approach can be applied [22]. For the probability of complete coverage of all haplotypes occurring with a frequency of at least ρ, we have *p*≥(1−*e* ^−*c*,ρ^)*^n^*. Since *c* = *NL*/*n*, where *N* is the number of reads and *L* is the read length, sequencing
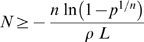
(1)reads will ensure that the completely consistent haplotypes assembled from the reads are an explaining set for these haplotypes. For example, in order to cover all haplotypes of 5% or higher frequency of length 1000 bases with reads of length 100 with 99% probability, at least 2302 reads need to be sequenced; to reach haplotypes at 1% frequency with 99% probability, 11,508 reads are needed. Notice that the number of reads needed scales linearly with the inverse of the smallest frequency desired. We note that the actual number of required reads can be much smaller in genomic regions of low diversity.

If the condition of the proposition is violated, we can remove the violating set of reads to obtain a new set satisfying the condition. This amounts to discarding reads that either contain mistakes in the error correction or come from haplotypes that are at a too low frequency in the population to be fully sequenced. Thus the resolution is inherently a function of the number of reads.

We are now left with finding a minimal explaining set of completely consistent haplotypes. Restricting to this subset of haplotypes reduces the computational demand of the problem significantly. The proposition implies that an explaining set of completely consistent haplotypes is precisely a set of paths in the read graph from the source to the sink, such that all vertices of the read graph are covered by at least one path. We call such a set of paths a *cover* of the read graph. The following result shows that a minimal cover can be computed efficiently (see Methods for a proof).

### Dilworth's Theorem [30]

1. Every minimal cover of the read graph has the same cardinality, namely the size of the largest set **Q** of vertices such that there are no paths between elements of **Q**.
2. A minimal cover of the read graph can be computed by solving a maximum matching problem in an associated bipartite graph. This matching problem can be solved in time at worst cubic in the number of irredundant reads.

The minimal path cover obtained from the maximum matching algorithm is in general not unique. First, it provides a minimal chain decomposition of the graph. A *chain* in a directed acyclic graph is a set of vertices that all lie on at least one common path from the source to the sink. A chain can generally be extended to a number of different paths. Second, the minimal chain decomposition itself is in general not unique. However, the cardinality of the minimal cover is well-defined. It is an important invariant of the set of reads, indicating the smallest number of haplotypes that can explain the data. Notice that the size of the minimal read graph cover can be greater than the maximum number of haplotypes in a given window of the error correction step. The cardinality of the minimal cover is a global invariant of the set of reads.

### Algorithm 2. (Minimal set of explaining haplotypes)

Input: A set **R** of aligned, error corrected reads satisfying the conditions of the proposition

Output: A minimal set of explaining haplotypes for **R**

Procedure:

1. Construct the read graph **G_R_** associated with **R**.
2. Compute a minimal chain decomposition of the read graph.
3. Extend the chains in the graph to paths from the source to the sink in **G_R_**.
4. Output the set of haplotypes corresponding to the paths found in step 3.

The algorithm can easily be modified to produce a non-minimal set by constructing multiple chain decompositions and by choosing multiple ways to extend a chain to a path. We note that the set of all paths in the graph is generally much too large to be useful. For example, the HIV datasets give rise to up to 10^9^ paths and in simulations we often found over 10^12^ different paths in the graph. Generating paths from minimal explaining sets is a reasonable way of sampling paths, as we will see below when discussing simulation results (see also Figure 4 and Figure S1).

Finally, if the conditions of the proposition are not satisfied, i.e., if the coverage is too low and the set of completely consistent haplotypes does not contain an explaining set, then that condition can be relaxed. This corresponds to modifying the read graph by adding edges between all non-overlapping reads. Algorithm 2 will then again find a minimal set of explaining haplotypes.

### Haplotype frequency estimation

A virus population is a probability distribution on a set of haplotypes. We want to estimate this distribution from a set of observed reads. Let **H** be a set of candidate haplotypes. In principle, we would like **H** to be the set of all possible haplotypes, but in practice we must restrict **H** to a smaller set of explaining haplotypes as derived from Algorithm 2 in order to make the estimation process feasible. Let **R** be the set of all possible reads that are consistent with the candidate haplotypes in **H**. The read data is given as a vector *u* ∈ **N^R^**, where *u_r_* is the number of times that read *r* has been observed.

**Figure 5:**
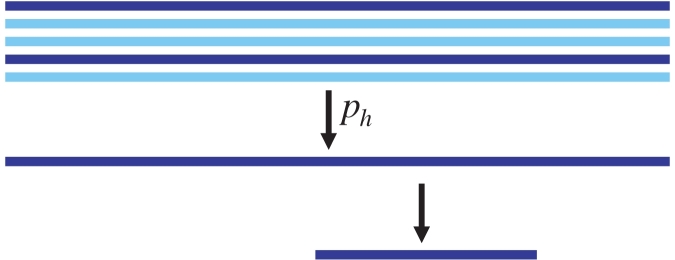
Schematic representation of the sampling process. The virus population is represented by five genomes (top) of two different haplotypes (light versus dark lines). The probability distribution is *p* = (3/5, 2/5). The generative probabilistic model assumes that haplotypes are drawn from the population according to *p* and reads are sampled uniformly from the haplotypes (bottom).

Our inference is based on a statistical model for the generation of sequence reads from a virus population. Similar models have been used for haplotype frequency estimation [31]–[33]. We assume that reads are sampled as follows (Figure 5). First, a haplotype *h* is drawn at random from the unknown probability distribution *p* = (*p_h_*)*_h_*_∈**H**_. Second, a read *r* is drawn with uniform probability from the set of all reads with which the haplotype is consistent. Estimating the structure of the population is the problem of estimating *p* from *u* under this generative model.

Let *H* be the hidden random variable with values in **H** that describes the haplotype and *R* the observed random variable over **R** for the read. Then the probability of observing read *r* under this model is
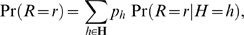
where the conditional probability is defined as Pr(*R* = *r*∥*H* = *h*) = 1/*K*, if *h* is consistent with *r*, and 0 otherwise. Here *K* is the number of reads *r* ∈ **R** that *h* is consistent with. Since we assume that all haplotypes have the same length, *K* is independent of both *r* and *h*.

We estimate *p* by maximizing the log-likelihood function
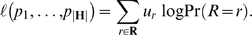
This is achieved by employing an EM algorithm (see Methods for details). Each iteration of the EM algorithm runs in time *O*(|**R**||**H**|). For example, for 5000 reads and 200 candidate haplotypes, the EM algorithm typically converges within minutes on a standard PC. Software implementing the algorithms for error correction, haplotype reconstruction, and frequency estimation is available upon request from the authors.

### Simulation results

We have simulated HIV populations of different diversities and then generated reads from these populations by simulating the pyrosequencing procedure with various error rates and coverage depths. The first 1 kb of the HIV *pol* gene was the starting point for all simulations. We separately analyze the performance first of error correction, then of haplotype reconstruction, then of haplotype frequency estimation, and finally of the combination of these three steps.

The simulations show that Algorithm 1 reduces the error rate by a factor of 30. This performance is largely independent of the number of haplotypes in the population (Figure S3). The program ReadSim [34] was used to simulate the error process of pyrosequencing. The error rate after alignment is about 1 to 3 errors per kb, so we are left with about 0.1 errors per kb after error correction. As the population grows and becomes more diverse, the alignment becomes more difficult resulting in a smaller error reduction (Figure S3).

In order to assess the ability of Algorithm 2 to reconstruct 10 haplotypes from 10,000 error-free reads (yielding about 1500 irredundant reads) we generate increasing numbers of candidate haplotypes. This is achieved by repeatedly finding a minimal set of explaining haplotypes until either we reach the desired number of haplotypes or we are unable to find more haplotypes that are part of a minimal explaining set.

**Figure 6:**
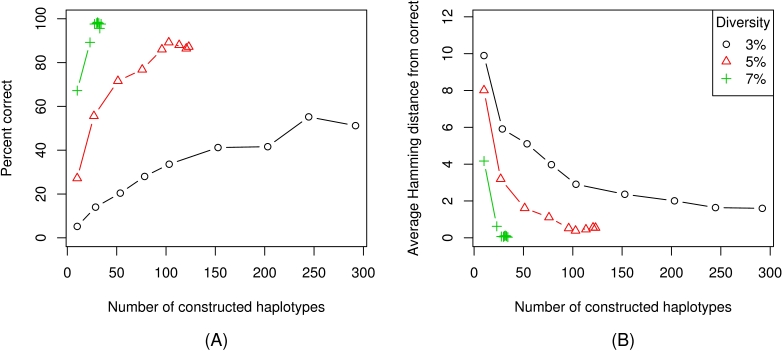
Haplotype reconstruction. Up to 300 candidate haplotypes were generated using Algorithm 1 from 10,000 error free reads drawn from populations of size 10 at varying diversity levels. Displayed are two measures of the efficiency of haplotype reconstruction: the percent of the original haplotypes with exact matches among the reconstructed haplotypes (A), and the average Hamming distance (in amino acids) between an original haplotype and its closest match among the reconstructed haplotypes (B).

Figure 6 visualizes the enrichment of recovered true haplotypes with increasing number of candidate haplotypes for different levels of population diversity. While in low-diversity populations exact haplotype reconstruction can be very challenging (Figure 6A), the algorithm will always find haplotypes that are close to the true ones. For example, at 5% diversity 10 out of 50 candidate haplotypes will match the original 10 haplotypes at an average Hamming distance of just 1.6 amino acid differences (Figure 6B). With larger populations, the performance is similar although more candidate haplotypes need to be generated (Figure S2). Given that the read graphs considered in this test had about 3·10^10^ total paths, the strategy of repeatedly finding minimal sets of explaining haplotypes is very efficient for haplotype reconstruction.

Figure 4 shows how the haplotype reconstruction problem gets harder at lower diversity. In each graph, the bottom five lines correspond to reads matching one of the original five haplotypes uniquely. The sixth line on top (if present) corresponds to reads that could come from several haplotypes. At 3% diversity (Figure 4A), only one of the haplotypes is reconstructed well. At 5% diversity (Figure 4B), the decomposition is almost correct except for a few small “crossovers”. At 7% diversity (Figure 4C), the chain decomposition exactly reconstructs the five haplotypes. By using multiple decompositions we can reconstruct many of the haplotypes correctly (Figure S1) even in low diversities.

**Figure 7:**
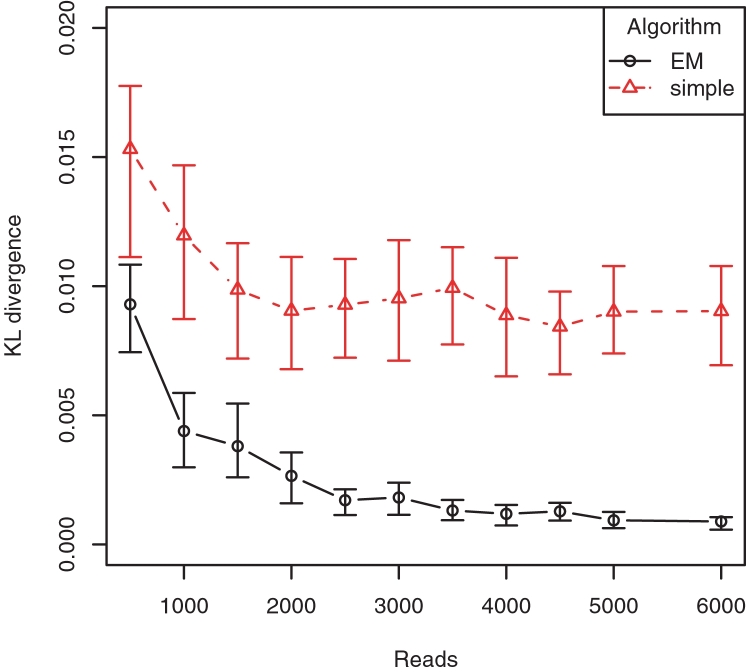
Haplotype frequency estimation. Haplotype frequencies were inferred using both the EM algorithm (circles) and a simple heuristic algorithm (triangles); the resulting distance from the correct frequencies is measured using KL divergence. Error bars give the interquartile range over 50 trials. The populations consisted of 10 haplotypes at equal frequency and 5% diversity. The input to the algorithms was a set of reads simulated from the population and the original 10 haplotypes.

The performance of the EM algorithm for haplotype frequency estimation described above is measured as the Kullback–Leibler (KL) divergence between the original population *p* and its estimate 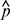. We consider populations with 10 different haplotypes, each with frequency 0.1, at 5% diversity. Haplotype frequencies are estimated from between 500 and 6000 error-free reads (Figure 7). The performance of the EM algorithm is compared to that of a simple heuristic method, which assigns frequencies to the haplotypes in proportion to the number of reads they explain (see Methods). For both methods, the KL divergence 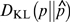 decreases roughly exponentially with the number of reads. However, the EM algorithm significantly outperforms the heuristic for all sizes of the read set and this improvement in prediction accuracy increases with the number of reads.

In order to test the combined performance of the haplotype reconstruction and frequency estimation, our basic measure of performance is the proportion of the original population that is reconstructed within 10 amino acid differences. This measure, which we call φ_10_, is defined as follows (see also Methods). For each inferred haplotype, we determine the closest original haplotype and sum up the frequencies of all inferred haplotypes that differ from their assigned original haplotypes by at most ten sites. This performance measure indicates how much of the population has been reconstructed reasonably well. It is less sensitive to how well haplotypes and haplotype distributions match (see Figures 6 and 7 for those performance measures).

**Figure 8:**
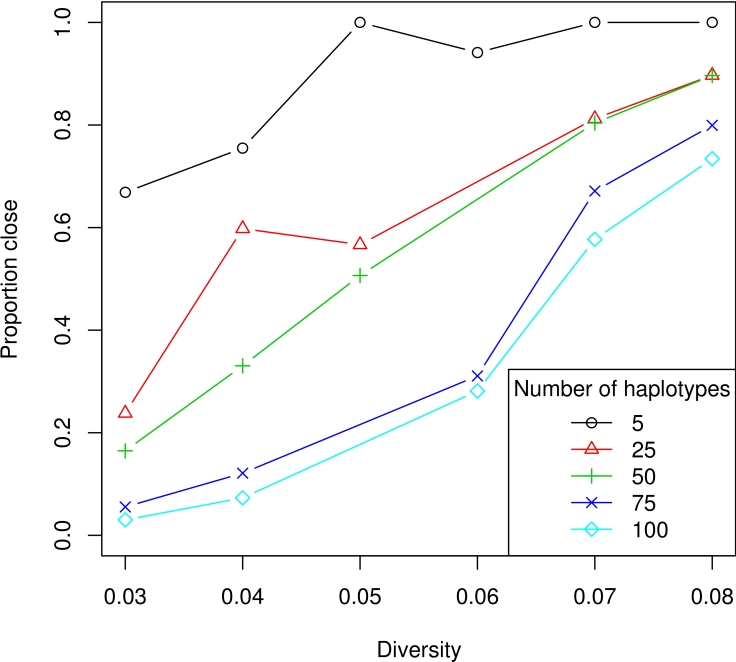
Combined population reconstruction procedure. The proportion of the population reconstructed within 10 amino acid differences (ϕ_10_, “proportion close”) is shown. Here 10,000 error-free reads were sampled from populations with diversity between 3 and 8% and with between 5 and 100 haplotypes of equal frequency. Haplotypes were reconstructed and then frequencies were estimated.

For the first simulation of combined performance, we consider error-free reads from populations consisting of between 5 and 100 haplotypes, each with equal frequency, at diversities between 3 and 8%. We simulated 10,000 error-free reads of average length 100 from these populations and ran haplotype reconstruction and frequency estimation. Figure 8 shows that performance increases as diversity increases and drops slightly as the number of haplotypes increases. As we saw above, we would expect to be able to reconstruct populations with size approximately 100 using 10,000 reads under the Lander–Waterman model.

**Figure 9:**
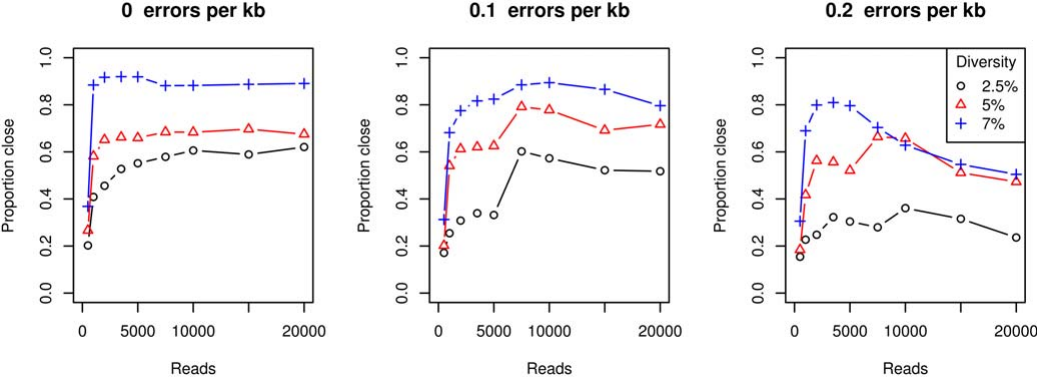
Population reconstruction with errors. Proportion of population reconstructed within 10 amino acid differences (ϕ_10_, “Proportion close”) using haplotype reconstruction and frequency estimation. The original populations had 10 haplotypes of equal frequency at varying levels of diversity. Error was randomly introduced in the simulated reads to mimic various levels of error correction.

For the second combined test, we tested all three steps: error correction, haplotype reconstruction, and frequency estimation. In order to model the miscorrection of errors, we ran ReadSim [34] to simulate the actual error process of pyrosequencing and then ran error correction. New, error-free reads were simulated and errors were added through sampling from the distribution of the uncorrected errors in order to reach error rates of exactly 0.1 and 0.2 errors per kb. Figure 9 summarizes the results of this analysis for 10 haplotypes at varying diversities. The combined procedure performs very well on error-free reads that are diverse enough. As errors are introduced, performance decreases; however the method still recovers much of the original population. For example, at 0.1 errors per kb, which is the error rate expected with current pyrosequencing technology and our error correction method (see above), as few as 3500 reads are required for approximately recovering 55% of a population of 5% diversity.

Figure 9 also indicates, for the datasets with error rate 0.2, a small performance loss as the number of reads increases. This phenomenon appears to be related to the fact that more reads give rise to more paths in the graph, thereby increasing the chances that completely consistent haplotypes that contain errors are assigned positive probabilities. In fact, the size of a minimal path cover increases approximately linearly with the number of reads and this increase does not appear to depend much on population diversity (Figure S4).

### Analysis of HIV samples

Our second evaluation of population reconstruction is based on ultra-deep sequencing of drug-resistant HIV populations from four infected patients [5]. The four virus populations were analyzed independently using pyrosequencing and clonal Sanger sequencing. Table 1 shows the resulting statistics on the datasets. The pyrosequencing based approach mirrors very closely the clonal sequencing. To compare the populations inferred from pyrosequencing to the clonal sequences, we use the measure φ_1_, which indicates the percent of the inferred population that matches a clonal sequence within one amino acid difference. This is used instead of φ_10_ used before in order to provide a more sensitive performance measure. In all samples, at least 51.8% of the inferred populations were within one amino acid difference of a clonal haplotype. Based on the present data, we cannot decide whether the additional inferred haplotypes went undetected by the Sanger sequencing, or if they are false positives of the reconstruction method.

**Table 1:** Population reconstruction from four HIV samples.

	Pyrosequencing				Haplotype reconstruction			Comparison to clonal seq.		
Sample	Reads	Irred.	ρ_99_	Gaps/kb	Err/kb	Min. cov.	Diversity	Clones	Avg. dist.	φ_1_
V11909	5177	641	2.2	3.3	1.10	22	15.8	65	1.81	51.8
V54660	7777	228	1.5	2.3	1.67	4	1.0	32	0.34	99.6
V3852	4854	227	2.4	3.4	1.33	7	1.4	42	0.29	100.0
V2173	6304	354	1.8	2.3	1.31	4	2.3	26	0.81	86.6

The first four columns describe the pyrosequencing data: the number of reads, the number of irredundant reads, the expected frequency (in percent) of the least frequent haplotype we can expect to cover with 99% confidence, and the number of gaps per kb in the aligned data. The next three columns describe the reconstruction algorithm: the number of non gap characters changed in error correction, the size of a minimal explaining set of haplotypes, and the diversity, measured as the expected number of amino acid differences among the estimated population. After error correction, reads were translated into amino acids. The last three columns describe the validation using (translated) clonal sequences: the number of clones sequenced, the average distance between the estimated population, and the closest Sanger haplotype, and φ_1_, the percentage of the estimated population that was close (up to 1 amino acid difference) to a clone.

We found many additional haplotypes in our analysis of the most complex sample, V11909. Table 2 shows a comparison between the inferred population for V11909 and the clonal haplotypes. The populations were analyzed at 15 positions in the protease associated with drug resistance, taken from the HIV Drug Resistance Database [35]. All but four of the 65 clonal haplotypes (6.1%) are matched in the inferred population, and the frequencies in the inferred population are a reasonable match to the frequencies of the mutation patterns in the clonal haplotypes. Using the Lander–Waterman model, we find that the pyrosequencing reads obtained from the HIV samples are enough to reconstruct with 99% probability all haplotypes that occur at a frequency of at least 2.2% (Tab. 1). By comparison, the Sanger sequencing approach yielded 65 clonal sequences, 37 of which were mixtures of two or more clones.

**Table 2:** Clonal Sanger sequencing versus pyrosequencing.

Frequency		
Sanger	Pyro	Mutations
52.3	19.3	M46I, I54V, G73I, I84V, L90M
12.3	19.0	M46I, I54V, G73S, I84V, L90M
9.2	9.4	M46I
6.2	5.6	
4.6	7.1	M46I, I54V, G73S, L90M
4.6	1.9	M46I, I54V, G73I, L90M
3.1	5.8	M46I, G73I, I84V, L90M
3.1	0.0	L33F, M46I, I54V, G73S, I84V, L90M
1.5	1.9	M46I, L90M
1.5	0.0	L33F, M46I, I54V, G73I, I84V, L90M
1.5	0.0	M46I, I54V, G73N, I84V, L90M
0.0	4.9	M46I, G73I
0.0	4.7	M46I, I84V
0.0	4.0	M46I, G73S, I84V, L90M
0.0	3.1	M46I, I54V, G73S, V82I, I84V, L90M
0.0	2.9	M46I, I54V, G73I, I84V
0.0	2.9	M46I, I50V, I54V, G73I, I84V, L90M
0.0	2.0	I84V
0.0	1.4	I54V, G73I, I84V, L90M
0.0	1.2	M46I, I50V, I54V, G73S, L90M
0.0	1.1	M46I, I54V
0.0	1.0	M46I, I50V, I84V, L90M
0.0	0.5	M46I, I54V, G73I
0.0	0.3	G73S, I84V, L90M

Displayed are the patterns of resistance mutation for the 65 Sanger sequences and the estimated population for sample V11909. Mutation patterns were restricted to 15 positions in the protease (amino acids 23, 24, 30, 32, 33, 46, 48, 50, 53, 54, 73, 82, 84, 88, and 90) associated with PI resistance.

## Discussion

Pyrosequencing constitutes a promising approach to estimating the genetic diversity of a community. However, sequencing errors and short read lengths impose considerable challenges on inferring the population structure from a set of pyrosequencing reads. We have approached this task by identifying and solving consecutively three computational problems: error correction, assembly of candidate haplotypes, and estimation of haplotype frequencies. Our methods focus on the situation where a reference genome is available for the alignment of reads. This is the case, for example, for many important pathogens, such as bacterial and viral populations.

The procedure consists of three steps. First, error correction is performed locally. We take windows of fixed width over the aligned reads and cluster reads within the windows in order to resolve the local haplotype structure. This approach is based on previous methods [27],[28] that are specifically tailored to pyrosequencing reads. Next, haplotypes are reconstructed using a new application of a classic combinatorial algorithm. This step is the main theoretical advance in this paper. Finally, haplotype frequencies are inferred as the ML estimates of a statistical model that mimics the pyrosequencing process. We have developed an EM algorithm for solving this ML problem.

Haplotype reconstruction is based on two assumptions: consistency and parsimony. We require that each haplotype be constructible from a sequence of overlapping reads and that the set of explaining haplotypes be as small as possible. The Lander–Waterman model of sequencing implies lower bounds on the number of reads necessary to meet the first requirement. The fundamental object for haplotype reconstruction is the read graph. A minimal set of explaining haplotypes corresponds to a minimal path cover in the read graph, and this path cover can be found efficiently using combinatorial optimization. Moreover, the cardinality of the minimal path cover is an important invariant of the haplotype reconstruction problem related to the genetic diversity of the population.

We believe that these methods are also applicable to many metagenomic projects. In such projects, estimation of the diversity of a population is a fundamental question. The size of a minimal cover of a fragment assembly graph provides an intuitive and computable measure of this diversity.

We have validated our methods by extensive simulations of the pyrosequencing process, as well as by comparing haplotypes inferred from pyrosequencing data to sequences obtained from direct clonal sequencing of the same samples. Our results show that pyrosequencing is an effective technology for quantitatively assessing the diversity of a population of RNA viruses, such as HIV.

Resistance to most antiretroviral drugs is caused by specific mutational patterns comprising several mutations, rather than one single mutation. Thus, an important question that can be addressed efficiently by pyrosequencing is which of the resistance mutations actually occur on the same haplotype in the population. Since our methods avoid costly clonal sequencing of the HIV populations for determining the co-occurrence of HIV resistance mutations [36], pyrosequencing may become an attractive alternative to the traditional clonal Sanger sequencing.

The sample size of approximately 10,000 reads we have considered provides us with the opportunity of detecting variants present in only 1% of the population. Pyrosequencing can produce 200,000 reads and thus twenty populations could be sequenced to a good resolution using a process less labor intensive than a limiting dilution clonal sequencing to a similar resolution of a single population.

The simulations suggest that the method works best with populations that are suitably diverse. Intuitively, the information linking two reads together on the same haplotype decays rapidly in sections of the genome where there are few identifying features of that haplotype (as in a region of low diversity). In particular, repeats of sufficient length in the reference genome can completely destroy linkage information. However, at some point the benefits of increased diversity will be partially reduced by the increased difficulty of the alignment problem. With more diverse populations or true indels, alignment to single reference genome will become less accurate.

The HIV *pol* gene analyzed here is on the low end of the diversity spectrum. The *env* gene with its higher variability may be a better target for some applications. We expect the proposed methods to improve early detection of emerging drug resistant variants [37],[38], and to support the genetic and epidemiological study of acute infections, in particular the detection of dual infections [39].

Since our computational procedure produces an estimate of the entire virus population, it allows the study of fundamental questions about the evolution of viral populations in general [40]. For example, mathematical models of virus evolution can be tested directly within the accuracy of estimated viral haplotype frequencies [41]. Predicting viral evolution is considered an important step in HIV vaccine development [10].

In addition to the promising biological applications, there are many interesting theoretical questions about reconstructing populations from pyrosequencing data. The errors in pyrosequencing reads tend to be highly correlated, as they occur predominately in homopolymeric regions. While this can make correction more difficult (a fact which can be counteracted by the use of quality scores), we believe that it can make haplotype reconstruction more accurate than if the errors were uniform. If errors are isolated to a few sites in the genome, fewer additional explaining haplotypes are needed than if the errors were distributed throughout. The exact relationship between the error process of pyrosequencing, error correction, and haplotype reconstruction is worthy of further study.

As pyrosequencing datasets can contain 200,000 reads, it is worthwhile to investigate how our methods scale to such large datasets. Haplotype reconstruction is the only step that is not immediately practical on such a large number of reads, since it is at worst cubic in the number of irredundant reads. However, problems of this size are approachable with our methods as follows.

The theoretical resolution of the algorithms depends on two factors: first, the ability to differentiate between errors and rare variants; and second, whether there are enough reads so that we can assemble all haplotypes. We have seen that the number of reads necessary for assembly scales with the inverse of the desired resolution: if *N* reads cover all haplotypes of frequency at least ρ, then *kN* reads are needed to cover all haplotypes of frequency at least ρ*k*. However, the resolution of error correction is at most the overall error rate as the number of reads grows; see Table 3.

**Table 3:** Resolution of viral haplotype estimation.

Number of reads	1,000	5,000	10,000	50,000	100,000	200,000
**Error resolution (%)**	3.00	1.20	0.90	0.50	0.42	0.37
**Reconstruction resolution (%)**	11.50	2.30	1.20	0.23	0.12	0.06

Displayed are the resolution of error correction (binomial test only) and of haplotype reconstruction as a function of the number of reads. The resolution of the error correction is defined as the smallest frequency of a mutation that will be visible over the background error rates; it is calculated with error rate ε = 0.0025 and significance level α = 0.001. The resolution of the haplotype reconstruction (derived from the Lander–Waterman model) is the smallest haplotype frequency expected to be entirely covered by reads. For small read sizes, haplotype reconstruction is the limiting factor (underlined in the table) but for over approximately 35,000 reads, error correction is the limiting factor.

The limited resolution of error correction combined with the elimination of redundant reads makes haplotype reconstruction feasible for large datasets. For example, error correction on 200,000 reads with ε = 0.0025 and α = 0.001 will erase all variants with frequency below 0.365 (Table 3; Methods). In order to have enough information to reconstruct these variants under the Lander–Waterman model, we would expect to need only about 30,000 reads. Furthermore, in regions of low diversity, many of the reads will be redundant and are thus discarded before building the graph. For example, with 30,000 error-free reads simulated from 275≈1/0.00365 haplotypes at 5% diversity, typically about 13,000 reads are irredundant. This number of irredundant reads is near the limits of our current implementation.

Current and future improvements to pyrosequencing technology will lead to longer reads (250 bp), more reads, and lower errors. However, in order for huge numbers of reads to be of great help in the ultra-deep sequencing of a population, the error rates must also decrease. The performance of our methods as read length varies is an important question, given the availability of sequencing technologies with different read lengths (e.g., Solexa sequencing with 30 to 50 bp reads) and the desire to assemble haplotypes of greater size (e.g., the entire 10 kb HIV genome).

Notice that haplotype reconstruction seems to be quite good locally (Figure 4 and Figure S1) in that many reconstructed haplotypes contain large contiguous regions where they agree with a real haplotype. However, the measures of performance considered in this paper all deal with the entire haplotype and ignore partial results of this type. This would seem to imply that longer reads will improve the reconstruction performance on a fixed length genome; new performance measures will have to be developed to analyze these problems.

## Methods

### Statistical tests for error correction

We use two statistical tests for locally detecting distinct haplotypes. The first test analyzes each column of the multiple alignment window. We write *d* for the number of reads that overlap this window. We ask if the observed number of mutations (deviations from the consensus base) exceeds our expectation under the null hypothesis of one haplotype and a uniform sequencing error ε. The probability of observing *x* or more mutations is given by the binomial distribution
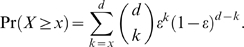
There are two parameters to set here: the error rate ε and the p-value α that is required for significance.

Next, we test pairs of mutations in two different alignment columns *u* and *v* using Fisher's exact test. The test statistic is the number *C* of co-occurrences, which under the null hypothesis of one haplotype follows the hypergeometric distribution
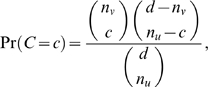
where *n_u_* and *n_v_* are the number of times the specific mutations have been observed in columns *u* and *v*, respectively [28]. Considering pairs provides more power if co-occurrences are observed on reads, but can not detect single mutation differences. We set the p-value for this test to be he same as for the binomial test.

The procedure tests all columns in the window using the binomial test and then all pairs of columns using Fisher's exact test. This can lead to over-counting of the number of haplotypes as follows. Suppose that in columns 1 and 2 the consensus base is A, but that there is a mutation C in some of the reads in each column. If both mutations are significant by themselves, this is evidence of three haplotypes in the window. If they are also significant together, this would be evidence of four haplotypes. However, there could be only two true haplotypes at these two positions: AA and CC. To correct for this, we subtract two from the count whenever two significant mutations are significant together and always occur on exactly the same set of reads.

We do not explicitly address the multiple comparisons problem associated with this testing procedure here and regard the significance levels of the tests as parameters of Algorithm 1. We account for the quality scores associated with each base by using (rounded) weighted counts in the test statistics. Gaps are treated as unknown bases and represented by a special character with quality score zero. We found that an error rate of 0.0025, a p-value of 0.001, and a window size of 24 provided the best error correction. These parameters can be tuned as follows. First, the window size should be chosen to best help the clustering. A large window provides more power since there are more identifying mutations, but also can be more difficult to cluster since many reads will only partially overlap the window.

Next, the p-value for the tests and the error rate should be adjusted to prevent false positives and negatives. The number of mutations required in a column before the mutation is considered significant can be calculated from the binomial distribution above. For example, with 10,000 reads of length 100 in a genome of length 1000, there will be approximately *d* = 1000 reads overlapping a small window. Setting ε = 0.0025 and α = 0.001, a mutation would have to occur nine times in a column to be significant according to the binomial distribution above. Thus, roughly speaking, the error correction would discard any mutations occurring in less than 9/1000≈1% of the population. Notice that this is quite similar to the estimate under the Lander–Waterman model (Equation 1), where 11,508 reads are needed to cover all haplotypes at 1% frequency.

Notice that the power of the error correction grows very slowly (Table 3). On a dataset with 200,000 reads, then error correction would eliminate any variants present in less than 0.365% of the population. However, only 30,000 reads are needed to achieve this resolution with haplotype reconstruction.

### Proof of Dilworth's Theorem

Suppose the read graph **G_R_** has *V* vertices and *E* edges. Since

**G_R_** is acyclic, it defines a partial order on the set of irredundant reads, **R**_irred_. Part (1) is then a direct application of Dilworth's theorem [30] to this partially ordered set. The associated bipartite graph has vertex set {**A**, **B**}, where both **A** and **B** are equal to **R**_irred_. There is an edge between *r* ∈ **A** and *s* ∈ **B**, if there is a path from *r* to *s* in **G_R_**. Then a maximal matching in the bipartite graph is equivalent to a minimal chain decomposition of **G_R_** [42].

For the time complexity, notice that building the read graph **G_R_** is of complexity *O*(*V* ^2^). Building the associated bipartite graph is equivalent to finding the transitive closure of the read graph and thus is *O*(*VE*). The efficient matching algorithm for the solution of the matching problem is due to Hopcroft and Karp [43]. For a general bipartite graph with *V*′ vertices and *E*′ edges, the Hopcroft-Karp algorithm is of time complexity 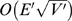. Since in our construction, *V*′ = 2*V* and *E*′ = *O*(*V* ^2^), the matching algorithm takes time *O*(*V* ^5/2^). Depending on the structure of the graph, either the transitive closure or matching problems can dominate, but both are of complexity *O*(*V* ^3^).

### EM algorithm for haplotype frequency estimation

We use an EM algorithm [44] to estimate the maximum likelihood haplotype frequencies. We iteratively estimate the missing data *u_rh_*, i.e., the number of times read *r* originated from haplotype *h*, and solve the easier optimization problem of maximizing the log-likelihood of the hidden model
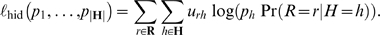
In the E step, the expected values of the missing data are computed as
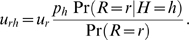
In the M step, maximization of ℓ_hid_ yields
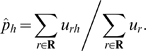



### Simulations

Our starting point is the first 1 kb of the wild type sequence of the HIV *pol* gene, encoding the 99 amino acids of the protease and the beginning of the reverse transcriptase. Random mutations are introduced into this strain in order to generate genetic diversity. We generated various populations in this way with diversities between 20 and 80 base pairs (2 to 8%). All haplotypes were set to have the same frequency in the population.

We report the expected value of the Hamming distance between two haplotypes drawn from a population as our basic measure of the diversity of a population. This statistic, which we call simply “diversity” can be thought of as a version of the Simpson measure [45] that takes into account the genetic structure.

We use ReadSim [34] (available from http://www-ab.informatik.uni-tuebingen.de/software/readsim/) to simulate the error process of pyrosequencing. We generate reads by running ReadSim with the options “–meanlog 0.15 –sigmalog 0.08 –filter” (aside from these options, we use the default parameters). This process results in about 7 insertions and 3 deletions per kb. Since pyrosequencing produces light coverage on the tails of the input genomes, we simulate by padding the region of concern with 100 nucleotides on each end and discard reads from the tails. The error correction (Algorithm 1) is run with window size of 24, p-value of 0.001, and error rate ε = 0.0025. We recorded the frequencies of errors at each position in the genome during simulations of populations of size 10 at 5% diversity. Sampling from these frequencies allowed us to create reads with precise error rates for Figure 9.

For the simulations of haplotype reconstruction, we generate pyrosequencing reads using the model described above and illustrated in Figure 5 complemented by uniform sequencing errors at rate 0, 0.1, and 0.2 per kb. We build the read graph and apply Algorithm 2 repeatedly until 200 candidate haplotypes are found. The EM algorithm was run with 10 random starting points. To speed up the EM algorithm, we round all frequencies *p_h_* < 10^−6^ to zero.

We also test a simple alternative to the EM algorithm as follows. For each haplotype *h* ∈ **H**, let *c_h_* count how many reads haplotype *h* is consistent with and set 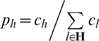. This estimatewill be correct under the given model if each read is consistent with exactly one haplotype. For evaluating the performance of the various steps of our reconstruction method, we use several basic measures of performance. To measure the distance between two sets of haplotypes (one original and one inferred), we calculate how many of the original haplotypes are found among the inferred haplotypes as well as the average of the distances between each inferred haplotype and its closest original haplotype (Figure 6). Distance is measured as Hamming distance on the amino acid level. To compare two populations with different frequencies but the same haplotypes, we use the Kullback–Leibler (KL) divergence *D*_KL_ (*p*∥*q*) = Σ*_h_*_∈**H**_ *p_h_* log(*p_h_*/*q_h_*), where *p* and *q* are the two discrete (haplotype) distributions with the same support **H** (Figure 7). To measure the performance of the entire process, we measure how much of the inferred population is close to the original population. Specifically, we calculate the percentage of the inferred population that is within a specified distance from one of the original haplotypes (Figure 9). We refer to this statistic as *φ_n_*, where *n* is the number of amino acid differences we allow.

### HIV sequence data

Virus populations derived from four treatment-experienced patients between 2000 and 2005 were sequenced using both pyrosequencing and limiting dilution Sanger sequencing. The plasma HIV-1 RNA levels in the four plasma samples were each greater than 100,000 copies/ml as determined using the VERSANT HIV-1 RNA assay [46]. Each sequence encompassed all 99 HIV-1 protease codons and the first 241 reverse transcriptase codons. The same genomic region of the same four samples was analyzed using limiting dilution and direct Sanger sequencing of the clones. Sample preparation and pyrosequencing and Sanger sequencing techniques are explained in detail in [5].

Briefly, ultra-deep pyrosequencing was performed on four RT-PCR products from RNA extracted from cryopreserved plasma samples. The median number of cDNA copies prior to sequencing was 100 with an interquartile range of 75 to 180. The resulting datasets consisted of between 4854 and 7777 reads of average length 105 bp. Reads were error corrected (Algorithm 1) and translated to amino acids. For haplotype reconstruction, Algorithm 2 was run repeatedly until all or at most 10,000 candidate haplotypes were found. The samples were translated into amino acids after the error correction step; thus, the haplotype reconstruction and frequency estimation algorithms are done on the amino acid level.

For the sample with the greatest diversity (V11909), the unamplified cDNA product was serially diluted prior to PCR amplification. Bidirectional sequencing was performed directly on 37 amplicons derived from the 1/30 cDNA dilutions and 31 amplicons derived from the 1/100 cDNA dilutions. Three sequences were discarded because of incomplete coverage. For the other three samples, we used the Sanger method to sequence a total of 32, 42, and 26 plasmid subclones per sample. Some of the sequences obtained from limiting dilutions contained mixtures of several clones. In this case, in order to measure the Hamming distance between an inferred haplotype and a clonal haplotype with ambiguous bases, we used the minimum distance over all possible translations of the ambiguous haplotype.

## Supporting Information

Figure S1 **Chain decompositions.**
Displayed are two different chain decompositions of the read graph for 1000 reads from a population of 5 haplotypes at 3% diversity. The bottom five lines correspond to reads matching a haplotype uniquely; the top to reads matching several haplotypes. One decomposition gets one haplotype entirely correct (top, black); the other gets two different haplotypes essentially correct (bottom, green and yellow). In this way, taking multiple chain decompositions allows us to reconstruct all haplotypes.
(0.84 MB EPS)

Figure S2 **Haplotype reconstruction.**
Up to 1000 candidate haplotypes were generated using Algorithm 2 from 10,000 error free reads drawn from populations of size 10, 20, and 50 (subfigures (A), (B), and (C), respectively) at varying diversity levels. Displayed is a measure of the efficiency of haplotype reconstruction: the average Hamming distance (in amino acids) between an original haplotype and its closest match among the reconstructed haplotypes.
(0.01 MB EPS)

Figure S3 **Error correction.**
Shown is the resulting error after error correction on populations with 4% diversity. Populations with up to 50 haplotypes of equal frequency were created. The program ReadSim was used to simulate pyrosequencing with an error rate of 3 to 6 errors per kb (after alignment). Error correction successfully reduced the error rate by a factor of approximately 30.
(0.02 MB EPS)

Figure S4 **Size of the read graph cover.**
Displayed is the computed lower bound on the population size from simulations with varying error rates and numbers of reads. Population diversity ranged from 3 to 7%. The lower bound is computed as the minimal size of a cover of the read graph. Error bars give interquartile ranges over 100 trials at different diversity levels. This estimated lower bound is quite accurate for error free reads; it seems to increase linearly with the number of reads if errors are introduced.
(0.02 MB EPS)
